# A Survey on Investigating the Need for Intelligent Power-Aware Load Balanced Routing Protocols for Handling Critical Links in MANETs

**DOI:** 10.1155/2014/138972

**Published:** 2014-03-23

**Authors:** B. Sivakumar, N. Bhalaji, D. Sivakumar

**Affiliations:** ^1^Department of IT, Adhiparasakthi Engineering College, Melmaruvathur, Tamil Nadu 603 319, India; ^2^Department of CSE, Tagore Engineering College, Chennai, Tamil Nadu 600127, India; ^3^Department of IT, Easwari Engineering College, Chennai, Tamil Nadu 600089, India

## Abstract

In mobile ad hoc networks connectivity is always an issue of concern. Due to dynamism in the behavior of mobile nodes, efficiency shall be achieved only with the assumption of good network infrastructure. Presence of critical links results in deterioration which should be detected in advance to retain the prevailing communication setup. This paper discusses a short survey on the specialized algorithms and protocols related to energy efficient load balancing for critical link detection in the recent literature. This paper also suggests a machine learning based hybrid power-aware approach for handling critical nodes via load balancing.

## 1. **Introduction**

A mobile ad hoc network (MANET) is a collection of wireless mobile hosts (or nodes) with limitations on energy utilization and forms a temporary network lacking fixed infrastructure. In an ad hoc network a message sent by a node reaches all its neighbouring nodes that are located at distances up to the transmission radius. Coverage and connectivity issues have a great impact on the performance of wireless networks. Optimized deployment strategy, sleep scheduling mechanism, and coverage radius reduce the cost as well as extend the network lifetime [1]. However, the network should be robust enough to sustain connectivity between nodes and maintain high quality services in the event of a failure. In addition to reliability, the quality of service such as the delay is also important. Mobile is battery powered and hence has limited lifetime. Due to excessive utilization a node may die which can result in energy depletion problem and thereby affects the overall network performance. Thus early detection and avoidance of energy depletion [2] is possible based on monitoring of power and remaining battery lifetime.

Critical links are weak links over which the communication cannot sustain over a period of time. This deterioration should be detected in advance and be rapidly switched to back up links such that the communicating mobile nodes are intact. One successful method is to maintain the smallest possible diameter of node links. However, due to mobility, frequent change in the formation of smallest diameter-weak links and the increase/decrease in such number are quite common and the solution to be suggested should withstand these challenges and is expected to preserve the critical links at a much faster pace. In this paper, we explore QoS challenges and perspectives for MANETs, survey the QoS mechanisms, and classify the state-of-the-art QoS-aware protocols related to critical link detection in the recent literature. This paper also proposes a link protection algorithm to be deployed in MANETs.

## 2. **QoS Requirements and Challenges**

QoS is the ability of a network to satisfy user and/or application requirements. Guaranteeing a certain QoS is a challenging issue due to the unpredictable and unstable wireless topology and severe resource (energy) constraints [3]. There are two main types of QoS provisions: hard QoS and soft QoS. Hard QoS should be provided with deterministic QoS guarantees which include strict bounds on packet delays, bandwidth, or packet losses. In soft QoS approach, the temporal violations on QoS guarantees are made flexible [4]. This becomes the escape point for critical nodes/critical links. But no point, by then, the network would have wasted enough of resources in creating and handling various communications.

### 2.1. **QoS Perspectives**

QoS perspectives can be of two types: application specific and network specific [5]. Application-specific perspective focuses on the quality of the application, such as lifetime [6, 7], coverage [8, 9], and deployment [10, 11]. Network-specific perspective like latency, packet loss, and reliability provides service quality during data delivery in the communication network [3].

### 2.2. **QoS Challenges**

Though many QoS challenges exist in traditional wireless networks, unreliability of links, severe resource constraints, and weak signal situations is the major criterion [12].

- * Resource Constraints*. Due to mobility and thereby frequent switching, MANETs lack of bandwidth, energy and processing capability. But these are adequate unlike those in wireless sensor networks (WSN), but that is a different issue. However, limited energy is the most crucial one since in many scenarios it is impossible or impractical to replace or recharge batteries of mobile nodes on the move, especially in public transportation in rural areas. Although energy harvesting via solar energy [13, 14] seems to be a promising solution to energy scarcity, present solar panels are still too large for mobile devices.
- * Node Deployment. *Unlike WSNs, the deployment of mobile nodes has to beat random. Therefore, neighbor discovery, path discovery, geographical information of the nodes, and clustering are the issues to be solved.
- * Topology Changes. *Changes in network topology might be due to mobility of nodes, failures in wireless links, improper functioning of mobile nodes, and depletion of energy and related constraints. In addition, sleep-listen schedules employed by power management protocols for energy saving also cause frequent topology changes. Inevitably, dynamic nature of the MANET topology introduces an extra challenge for QoS support.
- * Unbalanced Traffic*. Due to mobility and induced dynamism, sometimes amobile node may have too many data to handle and at times it may be not. Since there is no central entity to obtain an overall picture of what is happening within the underlying network, irregular traffic creates inefficient resource utilization. Smart routing protocols share load across routes but at the cost of a substantial overhead.

### 2.3. **QoS Performance Metrics**

- * Optimum Latency. *It is certain that, in order to minimize the end-to-end delay,the performance of routing layer should also be taken into account. However, the MAC layer may also come in handy to set optimised packet latency.
- * Contention Based and Contention Free Protocols. *Collision causesretransmission. This causes direct impact over the network performance. The amount of impact shall be measured by metrics like throughput, delay, packet delivery rate, and energy efficiency. Contention based protocols help us in preventing drastic collisions over the network. In the case of contention free protocols, adapting the time slots and setting the frequencies based on the network requirements would help much in terms of collision avoidance.
- *Maximizing Reliability. *To maximize reliability, collisions need to beminimized. ACK mechanisms and their variations can be used in identifying packet losses. Once a loss is identified in time, required retransmissions can be performed to fix the packet lost/altered under collision.
- * Minimizing Energy Consumption. *Reducing the consumed energy is the primerequirement because of the battery-limited operation of mobile devices. Power management protocols could come in handy at this juncture.
- * Handling of Parallel Transmission. *Since wireless media are shared inprinciple, it is not uncommon that packets are lost due to heavy interference. This cascades and reflects in the network performance which can be measured via throughput, delay, and energy efficiency. Practicing concurrent transmissions will put an end on the impact of interference caused due to parallel transmissions.
- * Change Management. *Node dynamism is a common scenario in MANETs. Often mobile nodes may deplete their energy and therefore get disconnected from the network. Similar to this, signal problems might also happen, but this in turn keeps the entire network inactive. In other words, if one mobile node struggles to receive signal, or is weaker in signal strength, as is the case for other nodes in the network, therefore this is not very serious. However, individual node disconnection results in routes disappearing between the nodes or new nodes might be added into the network which causes alteration in route setup. Links between nodes often change with respect to time due to environmental changes or topological changes or change in traffic patterns. Therefore, routing protocols should be very adaptive in learning the network dynamics.

### 2.4. **QoS Provisioning**

#### 2.4.1. **Power Control**

The main idea of power control is simply adjusting the transmission power based on the minimum power requirement for successful transmission [15]. Since it has the ability to control the network connectivity, reduction of energy consumption is a wiser option to QoS provisioning. In addition, this increases the concurrency in communications due to decreased interference and therefore improves the channel utilization. Implementing such power control mechanisms is not easy because of the very dynamic nature of wireless links and topologies

#### 2.4.2. **Clustering**

Due to existence of various levels of challenges in handling node mobility and load balancing, clustering of nodes becomes a serious difficulty. By establishing synchronization and coordination between nodes, clustering would become the reality. Clustering a set of mobile nodes improves internode connectivity and facilitates data aggregation. Therefore, clustering shall be used to provide support in terms of QoS, by means of power utilization and reliable communication. Clustering algorithms may be static or dynamic. Since mobile nodes are dynamic, static clustering of nodes would not be a wise decision; dynamic clustering adapts to change in network topology by changing or reconstructing the clusters. Change in the clusters is done via changing or rotating the cluster heads; this rotation of cluster heads happens with respect to the current topology prevailing in the network. Via clustering the load is evenly distributed among the nodes and, therefore, this ends in better power management. This is the reason for prevention of early battery exhaustion of cluster heads. However, this results in significant overhead due to message exchanges at various levels within and outside the cluster. But this cannot be avoided totally and has to be handled in a more intelligent manner.

#### 2.4.3. **Service Differentiation**

Service differentiation differentiates and prioritizes the traffic carried on the network based on one or more criteria and forms several traffic classes [16]. In this way, MAC layer treats each of these traffic classes differently by managing the resource sharing among them and tries to fulfill the requirements imposed by their degree of importance. Service differentiation consists of two phases: (i) priority assignment and (ii) differentiation between priority levels. In general, priority assignment schemes are categorized into the following three levels.

- * Static Priority Assignment*. Priority assignment is static if it never changes until the journey to destination, after once it is assigned. Static priority assignment depends on various network parameters like traffic class [17, 18], source type [19], and data delivery model [20].
- * Dynamic Priority Assignment*. In this scheme, the packet priorities may vary until delivery. This variable assignment scheme is beneficial in terms of frequent topology changes. The criteria for this scheme are remaining hop count, traversed hop count [18, 21], packet deadline [22], and remaining energy and traffic load [18, 23].
- * Hybrid Priority Assignment*. Unlike either static or dynamic, the good things in both schemes shall be merged together in hybrid priority assignment. However, balancing between static and dynamic priority schemes is to be done with utmost care to achieve better and reliable packet delivery. Sometimes, weights shall be assigned to choose between these criteria by which the degree of packet importance is calculated. This degree helps in mapping the packets to particular priority level and thereafter the priority assignment scheme shall take over. If priority assignment is complete without any partiality, resource sharing, the next phase of service differentiation will be taken care of by the underlying MAC layer based on the priority level, which has already indirectly encompassed the importance of packet data [3].

## 3. **The Critical Node Problem**

The efficiency of a network is highly dependent upon how the components are connected within the network. For this reason detecting critical nodes of a network is important to comprehend the topological characteristics and connectivity pattern of the network. It is also helpful in designing strategies for communication breakdowns in data communication networks. The critical node identification and minimization approach [24] can be used to solve the above-mentioned problems.

In critical nodes identification, for every node within a network, a subgraph is obtained by removing this node and all of its adjacent edges. And finally it is tested whether the subgraph formed is connected or not. If it is not connected, the corresponding node is a critical node [25]. As a result, the node having only one neighbor is not critical. Upon critical node identification, load sharing and resuming the normal traffic flow associated with that node are the two important things to be considered. Liu et al. [26] proposed a heuristic self-adapting inherit optimization algorithm to tackle the problem of load balancing on network and multiobjective route optimization with chaos group [27]. The authors [28] have proposed a dynamic route choice method of traffic engineering based on path measurement, bandwidth, and hops. However, all the methods mentioned above do not involve load problem of boundary link of the network which is of our prime concern since such links may upon performance deterioration tend to form the critical links.

As the number of nodes in the network increases, the chance of critical node failure also increases. Therefore, the ability to tolerate failure is equally important [8]. But this method is popular in mesh and torus networks only because of inherent path diversity provided by their topologies. The technique provided in the next section solves the mentioned problems and achieves fault tolerance for a variety of networks.

## 4. **Routing Protocols in MANETs**

Providing reliable high-throughput network connectivity to wireless clients is the most important property for wireless networks. Routing protocols satisfying specific network requirements have become the status symbol in the recent literature. Very less focus is being given to the improvement of existing routing algorithms [29]. We revisit routing protocols ad hoc on-demand distance vector routing (AODV) [30] and OLSR [31] and consider which properties should be carefully utilized and enhanced.

### 4.1. **AODV**

AODV stands for ad hoc on-demand distance vector routing. This on-demand routing is part of dynamic source routing (DSR). However, unlike DSR, which uses source routing, AODV takes a hop-by-hop routing approach. In addition, AODV combines the concept of destination sequence numbers from destination sequenced distance vector routing (DSDV). Since AODV is reactive in nature, it builds routes only as desired by source nodes. Therefore, nodes need not maintain routes to nonactive destinations. New route requests are handled in a quicker and easier manner than those in earlier routing protocols.

The route discovery of AODV is based on the exchange of RREQ (route request) and RREP (route reply) packets. For AODV with HOP, the shortest-hop path is preferred, and, hence, there is no need to process and forward later received RREQs with the same broadcast ID unless they have a higher sequence number. Duplicate RREQs that carry a better cumulative link metric value must be forwarded, so that all the possible routes are considered. AODV ensures link breakages and breakdowns are handled efficiently. AODV utilizes an RERR packet to alert the link disconnection [32]. When a link failure is detected and RERR is triggered, every node that receives the RERR removes the corresponding entry from its routing table and rebroadcasts it. Source nodes that use the broken link bootstrap the route establishment process by sending an RREQ.

As MANET routers are not stationary, link failures due to mobility are quite common. In addition, a link is disconnected and RERR is triggered when a transmission fails due to packet collisions, interference, or inadequate link rate selection, which often occurs temporarily. Although the route rediscovery process is crucial to maintain the end-to-end connectivity, unnecessary triggering of route recovery wastes network resources.

Caching the link metrics with every route requests would solve this problem to a certain extent. This demands forwarding of link metrics over route requests to every neighbor because which neighbor becomes the next hop is very much unknown. This action would end up with a situation where there are larger route request messages with increasing neighbors and eventually leads to a sizeable overhead in route setup.

Much similar to the significance of time to live filed (TTL), expected transmission count (ETX) is allowed for wireless networks. DSDV (destination-sequenced distance vector) and DSR (dynamic source routing) handle ETX to find paths with maximum throughput in multihop wireless networks [33]. However, maximum throughput is attained only approximately due to the frequently changing topology. The spanning tree variation of AODV, called AODV-ST (spanning tree) [34], combines AODV with the spanning tree algorithm. However, this is being applied in wireless mesh networks. Again, MANETs with high dynamism may find this algorithm a bit difficult due to overhead caused by fast computation. In other words, to adapt to changing topology, if MANETs should admit to increase in overhead, this algorithm may result in faster computation of routes. In addition, computing routes after addition of new nodes becomes a very common problem, and, therefore, employing AODV-ST would cost much in MANETs.

A good solution to the earlier problem is to use the tree based routing not compulsory but optional [29]. Recently, an efficient concept for multihop routing called opportunistic routing has been introduced in the literature [35, 36]. It has been, however, implemented for mesh networks. Studies have recorded an improvement in performance gain for this new concept when operating with multicast traffic. However, its contribution to unicast traffic is not that significant.

### 4.2. **OLSR**

In reactive (on-demand like AODV) routing approach, a routing protocol does not take the initiative for finding a route to a destination, until it is required. Since frequent topology change prevails as a major challenge in MANETs, proactive protocols are designed such that they immediately provide the route information as and when required through frequent periodic topology update. OLSR is proactive in nature. Therefore, here, every node maintains the topology information of the network. Every node runs the shortest path selection algorithm if any one of the following conditions is met: (1) a hello or TC message that conveys new link information is received; (2) a link is detected as not being a bidirectional link (during a predetermined time interval, hello messages have not been exchanged successfully in both directions); (3) a route to any destination expires. Since OLSR performs hop-by-hop routing, it supports nodal mobility which is the need of the hour.

### 4.3. **Offline Optimal Routing (MANUAL)**

This routing is partially constructed by the network administrators. In this routing, the optimality depends on the reference route set initially with respect to meeting several link quality criteria, determined by link metrics. This reference route needs to be optimal for the rest of the routing decisions to be optimal. Prior to the start of an active data session, every node collects the required link metric information over all the neighbouring links. With this information a node runs Dijkstra's algorithm to find the optimal route toward all destination nodes. A source node after knowing the optimal route compares the end-to-end metrics and selects the optimal path with the best end-to-end metric [29]. This protocol is proposed for wireless mesh networks; however, it may be applicable to MANETs also. The issue lies in establishing the reference route for MANETs. Intelligent load balanced routing protocols which learn quickly the near-optimal routes based on the traffic towards destination would be of much use.

### 4.4. **OSPF Extensions for MANETs**

Many routing protocols for MANETs have been developed such as OLSR [31] and AODV [30]. However, these mobile-only protocols attempt to divide a network where clear divisions no longer exist. The open shortest path first (OSPF) routing algorithm is a wired protocol that represents a good candidate to extend to support MANETs. OSPF has various features like support to multicast routing, multiple same-cost paths, and the ability to organize a network as a hierarchy [37]. OSPF protocol is destined principally for wired networks. Therefore, it works well with predictable topology changes under reliable medium. But these will not hold for highly dynamic and unpredictable MANET. The reason is that the nodes in a MANET tend to form peer relationships as they come into contact at random. This results in altering link state information, which will not be suitable for OSPF routing.

In addition, frequent packet loss exists due to path loss, interference, noise, shadowing, and multipath. Therefore, OSPF needs to be modified to meet the routing requirements of MANETs. This includes modifying the protocol to adapt to topology changes, minimizing data exchange for link state advertisements and control overhead [38].

Also, OSPF uses a link-state routing approach to find the least-cost path from a source router to destination routers within a group of routers. This exchange of routing information is called flooding. By flooding, at the expense of overhead, the routers are able to obtain a complete view of the network topology. The data exchanges are for advertising the state of links, missing as well as altered. This link state information is stored in link state database (LSDB). This database has the complete picture of the network topology and, therefore, is used as input to apply Dijkstra's algorithm for finding least cost paths from the routes to the other nodes which might become the chosen destinations. Since every router is associated with LSDB, there has to be a synchrony between LSDB of every router. This is done via periodic route broadcasts about the current state of links from every router.

In OSPF, the idea of designated router (DR) is extended for MANETs as MANET designated router (MDR) as a measure to reduce flooding overhead. Cisco's OSPF extension uses smart peer identification to handle the packets to reduce flooding overhead. This smart peer technique estimates a mobile node to be smart if it responds to a hello message within a marked/short span of time. For nodes which do not respond at the earliest, the size of hello message is reduced for the next round of hello message. This protocol is called incremental hello protocol. A better modification is OSPF multipoint relaying (MPR) which uses an MPR selector set to reduce adjacent nodes for which messages are broadcasted. Reportedly this modification of OSPF is the most advantageous in terms of reduction in flooding overhead [39].

## 5. **Protocols for Load Balancing in MANETs**

Over the years, several on-demand load balanced ad hoc routing protocols have been proposed. These routing protocols can generally be categorized [40] into three perspectives: delay based, traffic based, and combing the two. Delay-based protocols achieve load balancing by attempting to avoid nodes with high link delay. An example protocol is load-aware on-demand routing (LAOR) [41]. Traffic-based protocols achieve load balancing by evenly distributing traffic load among network nodes. Associativity based routing (ABR) [42], load balanced ad hoc routing (LBAR) [43], and traffic-size aware (TSA) scheme [44] fall under this category. In hybrid technique, load balancing is achieved by combining the features of traffic- and delay-based techniques. Examples are content sensitive load-aware routing (CSLAR) [45] and load-aware routing in ad hoc (LARA) [46].

Alternate path routing [47] provides load balancing by distributing the data traffic along the set of alternative paths. By using set of alternate paths, APR also provides failure protection. Therefore this protocol is only applicable for multichannel networks. Dynamic load-aware routing (DLAR) [48] makes use of the number of packets buffered as load in route selection. The mobile nodes attach their load information and broadcast the RREQ packet. To minimize the overlapped routes this protocol does not allow other intermediate nodes from replying to RREQ except the destination. If the route is congested, a new and lightly loaded route is selected to replace the overloaded path. Routes are hence reconstructed dynamically in advance.

Load-balanced ad hoc routing (LBAR) [43] is an on-demand routing protocol developed for delay-sensitive applications. LBAR defines degree of nodal activity to represent load on a metric node. The main idea here is to find optimal paths, which would reflect least traffic load, so that data packets can be routed with least delay.

Load sensitive routing (LSR) protocol [49] is based on the DSR protocol. This protocol utilizes network load information as the main path selection criterion. In LBAR and DLAR, destination nodes have to wait until the source identifies every possible route available. Rather, LSR does not require the destination nodes to wait for all possible routes. Instead, LSR can search for better paths dynamically if the active path becomes congested. Thus it uses a redirection method to find better paths effectively. This method can let the source node obtain better path without any increase in flooding cost and waiting delay on the destination nodes.

The above-mentioned routing protocols neither reflect burst traffic nor transient congestion. To solve this problem, weighted load-aware protocol (WLAR) [50] is proposed. This protocol selects the route based on the information from the neighbor nodes which are in the route to the destination. This protocol adopts basic AODV procedure and packet format. In WLAR, traffic load is defined as the product of average queue size of the interface at the node and the number of sharing nodes which are declared to influence the transmission of their neighbors. The average number of packets queued in interface is calculated by exponentially weighted moving average (EWMA).

In MANETs, since nodes have limited resources, the message overhead for load balancing is more critical. Simple load-balancing ad hoc routing (SLAR) [51] protocol attempts to reduce the overhead introduced by load balancing and is prevented from severe battery power consumption caused by forwarding packets. In SLAR, each node determines whether it is under heavy forwarding load condition, and in that case it leaves forwarding packets and lets some other nodes take that role.

Simple load-balancing approach (SLA) [52] tries to extend the expiration of mobile node power by preventing the traffic concentration on a few nodes, which may frequently occur under low mobility situations. This protocol resolves the traffic concentration problem by allowing each node to give up packet forwarding depending upon its own traffic load. However, there may be some selfish nodes that may deliberately give up packet forwarding to save their own energy, if an appropriate compensation is not given to them. Therefore, in SLA a credit-based scheme called protocol-independent fairness algorithm (PIFA) for urging nodes to voluntarily participate in forwarding packets is proposed.

Load-aware routing (LARA) [53] networks use traffic density (i.e., overall sum of traffic queue of node and its neighbors) to represent the degree of contention at the MAC layer. This density value is assumed from the self-maintained neighborhood table. The traffic queue of a node is defined as the average value of the interface queue length measured over a period of time. During route discovery, the destination selects the route with the minimum traffic cost.

Delay-based load-aware on-demand routing (D-LAOR) [41] protocol utilizes both the estimated total path delay and the hop count for route selection. The protocol can route around a congested node and thus can reduce the control overhead. In load-aware routing protocol (LARA) [54] a new metric for routing called traffic density which represents the degree of contention at the MAC layer is proposed. During the route setup, this metric is used to select the route with the minimum traffic load.

Correlated load-aware routing (CLAR) [55] protocol is a kind of on-demand routing protocol. Similar to traffic density of LARA, this protocol utilizes node traffic load as the primary route selection metric. The fundamental assumption is that the traffic load of a node depends on the traffic passing through this node as well as the traffic in the neighboring nodes. Since CLAR supports multipaths between the source and the destination, the destination node must select the best route among multipaths after analyzing the traffic load, the shortest hop distance, and the earliest path to arrive, respectively.

Most load balanced routing protocols measure the traffic size in number of packets. However, this is inaccurate since the size of the packets may differ. Load balanced routing through virtual path (LBRVP) protocol [44], an extension to the virtual path routing protocol (VPR) [46], uses the size of the traffic, through and around the network nodes, as the main route selection criterion. For any path that consists of multiple hops, the load metric of the path is the sum of all the traffic that is routed through all the hops that make up that path.

In any existing on-demand routing method, message transmission is initiated after forming the optimal route; however, successive message transmission occurs with particular nodes acting as routes when the network topology alteration is small. When network topology is relatively stable, the energy deficient nodes are included in the routing path, which could shorten the lifespan of the whole network. To solve this problem, a routing method which concerns power consumption rate is proposed. Energy consumption load balancing (ECLB) [56] makes balanced energy consumption available by calculating energy consumption rate of each node and choosing alternative route using the result to exclude the overburden-traffic conditioned node in route directory.

Instead of analyzing the history of traffic and deciding upon the packet routes, route decision based on traffic prediction would be more appealing. Prediction based adaptive load balancing (PALB) [57] is based on multipath routing protocol and traffic prediction. This protocol works on the assumption that several disjoint paths between source and destination node have been established by any existing multiple path routing protocols. The objective is to minimize traffic congestion and load imbalance by adaptively distributing the traffic among multiple disjoint paths based on traffic prediction. Source node periodically predicts the cross traffic of every node in the multiple disjoint paths. The packet distribution model distributes the traffic from packet filtering model across the multiple paths. The distribution of traffic is based on load balancing model which decides when and how to shift traffic among the multiple paths. The load balancing model operates based on evaluation of path.

Stability and measurement of path statistics. Therefore, the source node is able to adjust traffic distribution across multiple disjoint paths.

In energy-efficient load balancing protocol (EELBR) [58], the energy constrains related to routing protocols and workload balancing techniques are considered. This protocol employs adaptive load balancing technique to the MANET routing protocols with node caching enhancement. Hoang et al. [59] propose load balancing which is performed by the source node based on the probe packets sent by the destination node. Doing so will reduce the number of probe packets to half.

Workload-based adaptive load balancing (WBALB) [60] protocol makes each node react to the environment by analyzing their workloads. In this protocol, a node can chose to include or exclude itself from the forwarding activity. In other words, this protocol enables a node to join the route request (RREQ) forwarding action selectively. It utilizes interface queue occupancy and workload to make the selective forwarding decision. Each node maintains a threshold value (some arbitrary value initially) which varies according to the load status of a node. Overloading decreases the threshold value and vice versa. Periodically the node checks the threshold and, if it is found that the load has been low for a long enough period, its threshold value returns to the initial value. Immediately, the node is ready to handle additional communications until it gets overloaded.

Generally, the QoS of MANET is mostly affected by the congestion at any intermediate node in a selected routing path. Therefore, routing protocols handling congestion avoidance would be of high research interest. Congestion avoidance based load balanced routing (CALBR) [61] is a scheme designed to take route decisions considering the traffic congestion. In this protocol every node tracks the volume of packets successfully transmitted by self and by its one hop neighbors. Through this, the protocol attempts to select disjoint paths where there is no or less congestion.

Existing approaches try to improve the performance of routing protocols with respect to traffic balancing or energy consumption balancing.

In load balanced dynamic source routing (LBDSR) [62] the authors improve the well-known dynamic source routing (DSR) protocol to the so-called load balanced DSR (LBDSR) protocol. LBDSR shows better traffic balancing and energy consumption balancing, end-to-end delay, and route reliability metrics than DSR. In addition, LBDSR can also be customized to achieve better performance with respect to each of these metrics instead of being a trade-off between them. There is yet another variation to load balancing. Mechanisms [63] to push the traffic further from the center of the network to achieve load balancing would equally be a wise decision in route selection. Such protocols should take into account nodes' degree of centrality, for both proactive and reactive means of route selection decision.

AODV fundamentally does not support the quality of service (QoS) as well as load balancing. Pradeep and Soumya [64] propose some enhancements to AODV. The new protocol, referred to as Qos AODV, provides QoS and load balancing features by adding two extensions to the messages used during route discovery.

Distributed load balanced routing (DLBR) [65] is intended for a variety of traffic classes, fundamentally multimedia and nonmultimedia traffic, to establish the best routing paths. Multimedia traffic is given the highest priority. The algorithm calculates the cost metric on the basis of the load on the links. In the presence of high priority traffic, the routing of high priority traffic is performed over the lightly loaded links. In addition, the resources can be shared between the high priority traffic's path and low priority traffic.

The Fibonacci multipath load balancing protocol (FMLB) [66] is yet another distribution based load balancing approach where transmitted packets are distributed over multiple paths using the Fibonacci sequence. Such distribution increases the delivery ratio because the congestion is reduced by the distribution activity. The overhead lies in balancing the packet transmission over the selected paths and ordering them according to hop count. However, the shortest path is used frequently more than the other ones.

Node centric load balanced routing protocol (NCLBR) [67] is similar to how AODV operates. There are three distinct roles for nodes in NCLBR protocol, namely, terminal (nodes that are connected to the rest of the network by a single link), trunk (nodes that connect two different network segments), and normal (nodes other than trunk or terminal). Here each node itself avoids congestion in a greedy manner. It is the node's responsibility to divert congestion away from itself onto other alternative paths that may exist in the network. The main objective here is to avoid new path formation through a congested node.

In dynamic congestion detection and control routing (DCDR) [68], the routing decision is based on the estimations of the average queue length at the node level using which the current congestion level shall be detected. Based on this, every node generates a warning message to its neighbors. It is then the responsibility of neighbors to locate a congestion-free alternative path to the destination. Since congestion free route selection involves the neighboring nodes as well, this protocol ensures reliable communication within the MANET. However, high coordination is required between the nodes to operate at reduced overhead due to alternate path finding.

Ant based algorithm for load balancing (AntLBR) [69] is more robust as routing information is based on direct measurement of real-world network situations. This algorithm is hybrid in nature, that is, proactive some time and reactive other time. Data packets are routed stochastically according to pheromone tables (which are stored locally at each node). Each node also maintains a neighbor node table to keep track of nodes having wireless links. Load balancing is achieved by making use of multiple paths and then selecting optimal path mostly for transmission of packets from source to destination.

## 6. **Protocols for Critical Node Detection**

The communication networks having different types of nodes, links, and other resources cause uneven distribution of the traffic loads. Also the chances of failure increase with the growth of the network size. To avoid such uneven traffic distribution and failure and to continue with the usual traffic load, some precautions must be taken.

Critical rings [70] are conceptual rings formed around critical nodes to relieve the networks from such unequal traffic distribution and failure. A critical ring is defined as the nearest ring around a critical node by considering the nodes directly connected to that node, which may require inserting one or more links. Repeated formation of critical rings may eventually convert the critical node into noncritical node and can make the network fault tolerant. These critical nodes shall also be detected well in advance by critical node prediction [71]. Alternatively, critical nodes compensation algorithm [2] is also proposed in order to prevent network from partitioning, thereby insuring network connectivity and throughput.

DFS was equally used to detect critical nodes [72]. The algorithms in [73] require that a node should be aware of global topology. In practice, this method is inefficient and involves huge communication overhead. Distributed algorithms for critical node detection algorithm for wireless ad hoc networks greatly reduce communication overheads and the speed of detection [2].

Clustering the nodes is a wiser solution to reduce overhead and equally to achieve lesser critical nodes, but, here, a significant source of overhead is the reclustering of nodes due to dynamic changes in network topology. If we manage to minimize the effect of reclustering then we expect the performance of the network to be improved (e.g., more scalable and survivable network). One solution is to group the nodes by some operating mobility characteristics (like the direction of mobility, velocity, and location) and then to cluster them accordingly [74]. By this approach, the topology changes will not affect the intracluster connectivity. However, this grouping has to be very dynamic in nature for efficient reduction of communication overhead.

In the literature, there exist many network design methods to enhance the reliability and the performance by adding links to an existing network. There is a problem for determining a set of edges to meet the required edge connectivity or vertex connectivity by adding edges to a graph [75–77]. For considering path length, there is a problem for determining a set of edges to meet the required diameter by adding edges to a graph [78–80]. Chordal graph completion problem, interval graph completion problem, and Hamilton graph completion problem determine a set of added edges to make a given graph, a chordal graph, an interval graph, and a Hamilton graph, respectively [78]. Kamiyama and Miwa [81] deal with the measure to evaluate both the reliability and the performance and Katayama et al. [82] proposed an approximation algorithm for a problem to enhance both the reliability and the performance based on the measure by adding links to an existing network.

## 7. **Discussion**

### 7.1. **Open Challenges**

The open challenges faced by MANETs according to Shivasankar et al. [2] are bandwidth constraints, location dependent contention, quick route reconfiguration, loop free routing, reliability based on signal strength, residual battery power, minimize power consumption per packet, availability, and residual capacity.

To conserve battery energy of the nodes, there are various routing algorithms and schemes designed to select alternative routes. These algorithms and schemes are collectively known as “power-aware routing protocols.” To conserve energy, we would like to minimize the amount of energy consumed by all packets traversing from the source node to the destination node. Conserving energy neglects power consumption at individual nodes, which speeds up network partition by draining batteries of the critical nodes in the network one by one. The route between the partitions must go through one of these critical nodes. Therefore early identification and strengthening of critical node is a major issue to be tackled in a timely manner.

As a solution, we adapt the idea of link protection for mobile ad hoc networks. Kim et al. [29] observe that unexpected protocol behavior and inaccurate design of link metric cause nonoptimal route construction based on which the end-to-end performance is degraded. Interference generated by background traffic or from other sources can disturb the optimal route construction, thus making the resultant performance even worse. Accordingly, there is a need to design robust protocol operation and accurate link quality metric which are able to be unaffected by interference, thus building the optimal route in a robust manner. The link protection approach [83] aims to find the smallest number of links to be protected such that the diameter of the resulting graph by the failures of nonprotected links is less than or equal to a given integer. This problem is important not only from a theoretical viewpoint, but also from a practical viewpoint, as ISPs can enhance the reliability of their networks by protecting the small number of the critical links whose failures significantly deteriorate the performance in a network.

Alternatively, in a MANET the mobile nodes shall be clustered under various cluster heads. But this clustering should be performed considering the remaining battery power. However, due to heavy traffic, cluster heads close to base station may deplete their energy at a faster rate. This is popularly known as “hot spot” problem. Bagci and Yazici [84] propose a fuzzy energy-aware unequal clustering algorithm (EAUCF) that addresses the hot spots problem. EAUCF aims to decrease the intracluster work of the cluster heads that are either close to the base station or have low remaining battery power. A fuzzy logic approach is adopted in order to handle uncertainties in cluster-head radius estimation. A similar idea is yet to be applied in MANETs too to preserve nodes from becoming critical or to recover nodes that are in critical state.

### 7.2. **Desirable Features**

The following are the desirable features expected to be present in MANET protocols:

1. reducing flooding and flooding overhead;
2. minimising packet drop/congestion or increasing packet delivery rate;
3. reduced rate of energy depletion;
4. acceptable load balancing;
5. foresight into traffic patterns to plan for packet delivery/better traffic management;
6. intelligent and context aware protocol to predict QoS guarantees in spite of dynamic topology.

In this context, in future, we have plans to implement an intelligent routing protocol which is capable of clustering the mobile nodes in spite of QoS uncertainties to identify the set of critical links. An investigation of remaining energy conserved in the nodes with critical links would reveal the actual critical nodes that are to be really protected. Applying machine learning over the region of critical nodes for load balancing would serve as a profitable measure to prevent the critical nodes from energy depletion and, thereby, would improve the performance of the MANET.

The protocol we have designated for handling power-aware load balancing is aimed at the following dimensions.

1. Fundamental packet delivery is done via standard OSPF.
2. Mobile nodes are clustered by learning their frequency and direction of packet exchanges. Nodes that handle the same pattern of traffic are clustered together. This technique is known as self-similarity based clustering.
3. The packet forwarding by a node differs by the presence of that node in number of clusters. If the node is present in more than one cluster, the node cannot become a cluster head.
4. Cluster heads are generally chosen based on the loyalty of existence within the network.
5. To enable the protocol not to deplete the cluster head, we also used energy aware unequal clustering as an addition to self-similarity based clustering and therefore hot spot problem is avoided.
6. Handling of traffic between clusters is by ant colony algorithms.

Though hot spot problems are avoided; the approach we suggested had limitations on selection of cluster heads. Cluster heads would obviously need some reward for being a cluster head. If reward scheme is introduced, nodes would benefit by some means for servicing the cluster. Also, dynamism is totally unavoidable in MANETs and, therefore, introducing trust between nodes would be a welcome decision if the mobile nodes reappear frequently after been missing for shorter time periods.

## 8. **Conclusion**

The critical links are weaker links whose failures significantly deteriorate the performance in a network. These links must be detected well in advance to maintain mobile network connectivity. Graph algorithms could well be applied in early detection of critical links. In addition, we observe the need for load balancing around the critical nodes soon after they are located. This might have a greater contribution in two significant perspectives: (1) speeding up the network traffic which had been otherwise deteriorating due to the enormous load handled until then by the critical node; (2) improvising the throughput and reliability of the critical node by recovering it from critical state. We have applied machine learning algorithms for clustering the nodes based on energy efficiency and performing a load balancing. In future, prediction of network traffic patterns and applying the Bayesian belief networks over the clusters to decide upon trust parameters would be of our interest.
